# Analysis of current situation and influencing factors of cognitive dysfunction associated with type 2 diabetes and follow-up study on treatment effectiveness

**DOI:** 10.3389/fneur.2024.1419017

**Published:** 2024-08-16

**Authors:** Minli Liu, Zhiguo Wang, Jiming Han, Zhenzhen Mu, Hongyan Bian

**Affiliations:** ^1^Medical College of Yan'an University, Yan'an, China; ^2^Shanxi Provincial People’s Hospital, Taiyuan, China; ^3^Traditional Chinese and Western Medicine Geriatric Department/Health Department, Yan’an People’s Hospital, Yan’an, China

**Keywords:** cognitive impairment, influencing factors, insulin, metformin, type 2 diabetes mellitus

## Abstract

**Background:**

Many studies have explored the risk factors associated with cognitive impairment in patients with Type 2 diabetes mellitus (T2DM). However, research on determining the optimal threshold for these risk factors and comparative studies on the therapeutic effects of insulin and metformin is limited. This study aims to establish the optimal threshold for cognitive impairment risk factors in T2DM patients and compare the efficacy of insulin and metformin in treating mild cognitive impairment (MCI).

**Methods:**

A total of 308 patients with T2DM were included. The optimal threshold for cognitive impairment risk factors was determined using receiver operating characteristic curve and binary logistic regression models. MCI patients were divided into three groups: insulin, metformin, and insulin with metformin. The treatment effect was evaluated after a 6-month follow-up.

**Results:**

The study identified several factors that influenced cognitive function in T2DM patients, including female gender, duration of diabetes >13.50 years, years of education >7.50 years, and serum sodium level > 141.90 mmol/L. Metformin and insulin with metformin showed superior therapeutic effects compared to insulin alone, but no difference was observed between metformin and combination therapy.

**Conclusion:**

Special attention should be given to female and those with diabetes duration >13.50 years, as well as to individuals with educational level ≤ 7.50 years and serum sodium concentration ≤ 141.90 mmol/L. Metformin and insulin with metformin effectively improve MCI in patients with T2DM and outperform insulin monotherapy. The efficacy of metformin and combination therapy was found to be comparable.

## 1 Introduction

Type 2 diabetes mellitus (T2DM) is a chronic metabolic disorder characterized by high blood sugar levels due to insufficient insulin production or insulin resistance. The global prevalence of diabetes according to the 2021 data is an estimated 537 million people, a 16% increase from the previous year, representing 10.5% of the global population. By 2045, this number is projected to rise to 783 million (1).

Cognitive impairment in diabetes, also known as diabetes-related cognitive decline (2), refers to the alteration in cognitive functions in individuals with diabetes. Studies have shown that people with diabetes are 1.5 to 2.0 times more likely to experience cognitive impairments than non-diabetic individuals, and there is a significant association between diabetes and the decline in memory, language, and executive function (3). The annual incidence rate of dementia in individuals with mild cognitive impairment (MCI) in the general population ranges from 0.2 to 3.9%, whereas in T2DM patients, it ranges from 6 to 25%, which is much higher than that observed in the general population (4). China’s diabetes population has specific characteristics that differ from those in the West, such as an earlier age of onset and a higher incidence rate among people aged 30–40 years old. Most patients are aged 45–64 years. Additionally, β-cell dysfunction occurs earlier and more severely in China’s diabetes population compared to the West. Based on these two points, the risk of cognitive impairment in diabetes patients also increases. Therefore, studying the influencing factors of cognitive impairment in T2DM patients and exploring treatment methods for cognitive impairment patients is particularly important.

In T2DM patients, cognitive impairment can be attributed to various factors, including insulin resistance, inflammation, redox imbalance, cerebral microvascular dysfunction, dysbiosis of gut microbiota, disruption of metal homeostasis (e.g., calcium and iron), and lymphatic system dysfunction (5). A meta-analysis of 17 studies indicated that the incidence of cognitive impairment in elderly with diabetes was 48%, and another study estimated an prevalence of MCI in T2DM patients at 45.0%. The prevalence rate was found to be lower Europe compared to Asia, with higher rates observed among female and those aged over 60. Additionally, elderly diabetes with low education levels, living alone, or with a monthly of less than 2000 yuan exhibited high rates cognitive impairment (6, 7).Some researchers have also identified specific risk factors for cognitive dysfunction in T2DM patients, including macrovascular disease, microvascular complications, poor glycemic control, duration of diabetes, and elevated levels of triglycerides and total cholesterol (8, 9). For instance, Sun et al.’s research demonstrated that the longer the duration of diabetes and the lower the education level, the higher the risk of cognitive dysfunction among patients (8). However, there is a scarcity of studies exploring the specific thresholds at which these factors significantly increase the risk of cognitive dysfunction.

Research studies have provided insights into the potential benefits of intranasal insulin treatment for individuals with diabetes, including improved learning and memory functions, enhanced hippocampal neurogenesis, and increased brain insulin levels (10). A study involving amnestic MCI or mild to moderate Alzheimer’s disease (AD) patients found that daily administration of 20 IU regular insulin was associated with better story recall ability compared to the placebo group (11). However, a randomized double-blind clinical trial did not support the use of intranasal insulin in improving cognitive function in MCI and AD patients (12). Samaras et al. reported that patients taking metformin experienced a slower decline in executive function and overall cognitive decline, while those not taking metformin had an increased risk of developing dementia (13). Kodali et al. reached a similar conclusion, suggesting that initiating metformin treatment in late middle age can improve cognitive function in the elderly, possibly through inhibition of microglia activation, reduction of pro-inflammatory cytokine levels, and enhancement of hippocampal autophagy (14). Despite these findings, Porter et al. proposed that taking metformin may increase the risk of cognitive impairment, potentially due to a deficiency of B vitamins (15). Wu et al.’s longitudinal study found no association between the use of metformin and longitudinal memory changes in T2DM patients with MCI (16). Most studies have indicated that both insulin and metformin can improve cognitive function in patients, but there is a lack of research comparing the therapeutic effects of different drugs.

In this study, we aimed to evaluate the cognitive function of T2DM patients using the Mini-mental State Examination (MMSE). Our goal was to investigate the current status of cognitive dysfunction in T2DM patients and to explore the best cutoff values for risk factors associated with cognitive dysfunction. Additionally, we compared the therapeutic effects of insulin, metformin, and the combination of insulin and metformin on T2DM patients with MCI. The findings from this study will provide a foundation for early assessment and clinical intervention for T2DM patients with cognitive impairment.

## 2 Methods

### 2.1 Study population

This study selected patients admitted to the Endocrinology Department of Yan’an University Affiliated Hospital and the Comprehensive Internal Medicine Department of Dongguan Branch from December 2022 to December 2023 as the research subjects.

#### 2.1.1 Inclusion criteria

(1) Compliance with the diagnostic criteria for diabetes as formulated in the 2020 guidelines for the prevention and treatment of T2DM in China; (2) informed consent and voluntary participation in the study.

#### 2.1.2 Exclusion criteria

(1) Degenerative diseases such as AD, frontotemporal lobe dementia, Lewy body dementia, Parkinson’s disease dementia, and Huntington’s disease dementia; (2) Vascular diseases such as infarct dementia, subcortical arteriosclerotic leukoencephalopathy, and cerebral hemorrhage; (3) Cranioencephalic injury; (4) Infectious diseases such as multiple sclerosis dementia, human immunodeficiency virus disease, dementia caused by specific or non-specific infections, syphilis infection, and progressive multifocal white matter encephalopathy; (5) Congenital intellectual impairment; (6) Cerebrovascular functional disorders, mental disorders, intracranial tumors, etc.; (7) Malignant tumors; (8) Combined visual, hearing, and physical impairments; (9) History of ethanol addiction and drug abuse; (10) Genetic diseases that affect cognitive function, such as familial Alzheimer’s and hereditary multiple cerebral infarction dementia.

#### 2.1.3 Drop-out criteria

(1) non-compliance with medication regulations; (2) self-administration of other medications during the treatment period; (3) lost follow-up: This refers to situations where attempts to contact participants are unsuccessful, including cases where the phone number provided was invalid, the phone was turned off, or no one answered after multiple attempts made at different time periods. It can also include cases where the participant has passed away.

The Ethics Committee of Yan’an University Affiliated Hospital approved this study (YA-L20220014). All participants provided written informed consent in accordance with the Helsinki Declaration prior to their involvement.

### 2.2 Data collection

The researchers collected demographic data and clinical characteristics of all patients, including age, gender, education level, marital status, income, smoking history [defined as continuous or cumulative smoking for more than 6 months (17)], drinking history (defined as consuming alcohol at least once a week within a year, and currently drinking or abstaining from alcohol for less than 3 years), lifestyle, body mass index (BMI) (calculated as weight (kilograms) divided by height (meters) squared), systolic blood pressure (SBP), diastolic blood pressure (DBP), fasting plasma glucose (FPG), complications, and comorbidities. Participants should collect the following blood samples after fasting for a minimum of 6 h and refraining from drinking for at least 4 h. The blood samples were used to measure various parameters, including glycosylated hemoglobin (HbA1c), blood lipids (such as serum triglycerides (TG), total cholesterol (TC), low-density lipoprotein cholesterol (LDL-C), high-density lipoprotein cholesterol (HDL-C)), liver function indicators (such as albumin (ALB), globulin (GLB), albumin/globulin ratio (A/G), total bilirubin (TBIL), direct bilirubin (DBIL), indirect bilirubin (IBIL)), renal function indicators (such as Cys-C, β2 microglobulin (β2-MG)), electrolytes (such as sodium, glucose (GLU)), and inflammatory markers (such as C-reactive protein (CRP), fibrinogen (FIB)).

### 2.3 Cognitive testing

The cognitive function of all participants was evaluated using MMSE, which was developed by Folstein et al. (18) in 1975 and is widely used in clinical practice for diagnosing cognitive decline and dementia. The MMSE consists of five dimensions: orientation, memory, attention and calculation, recall, and language abilities. A total score range of 0 to 30 is used, with scores of 27–30 indicating normal cognitive function, 21–26 indicating MCI, scores of 10–20 indicating moderate cognitive impairment and ≤ 9 indicating severe cognitive impairment. The evaluations were conducted by researchers in a quiet environment.

### 2.4 Treatment plan

The treatment plan for patients with MCI involved dividing them into three groups: an insulin treatment group, a metformin treatment group, and an insulin-metformin treatment group, based on the discharge medical order records.

The insulin group received subcutaneous injections of Mendong insulin three times per day (before meals), starting with a dose of 0.6 U/kg. The dose was adjusted based on blood glucose level monitoring and medical advice. The specific drug used was Mendong Insulin Injection, manufactured by Novo Nordisk (China) Pharmaceutical Co., Ltd. It came in a 3 mL pen refill with 300 units and was approved by the National Pharmaceutical Approval Letter S20153001.Alternatively, patients in this group could receive subcutaneous injections of Degu insulin, with a dosage range of 0.3–0.5 units/kg once a day before bedtime. Again, the dosage was adjusted according to blood glucose level monitoring and medical advice. The Degu insulin used was manufactured by Novo Nordisk (Denmark) Pharmaceutical Co., Ltd., with specifications of 3 mL: 300 units (Changchong) and was approved by the National Pharmaceutical Approval Letter J20171096.

The metformin group was administered 1.5 g of metformin hydrochloride tablets orally once a day. The specific drug used was Metformin Hydrochloride Tablets produced by Shanghai Shiguibao Pharmaceutical Co., Ltd. from China and the United States. Each tablet had a specification of 0.5 g and was approved by the National Medical Approval Letter H20023370.

The insulin-metformin treatment group received a combination of the above two drugs.

After 6 months of treatment, cognitive function changes were assessed through phone follow-ups in each group. The MMSE was used to record and compare the baseline and end-of-treatment cycle results.

### 2.5 Statistical analysis

For statistical analysis, SPSS 25.0 software was utilized. For normally distributed data, mean ± standard deviation was used to describe the data. Data that did not follow a normal distribution were described using the median (quartile) [M (P25, P75)], while count data were described using frequency (n) and composition ratio (%). Univariate analysis of factors influencing cognitive dysfunction in T2DM patients was conducted using independent sample t-tests, Mann–Whitney U-tests, and χ2 tests. The optimal cutoff value for statistically significant factors identified in the univariate analysis was determined using ROC curves, with evaluation based on the area under the ROC curve, sensitivity, specificity, and Youden index. Multivariate analysis was performed using binary logistic regression analysis. The differences in Baseline data among different treatment groups were compared using analysis of variance (ANOVA), Kruskal-Wallis rank sum test, and chi-square test. The total score of MMSE and the scores of each dimension in each treatment group were compared using Wilcoxon signed-rank sum test, Kruskal-Wallis rank sum test between the groups, and the α split method was used for multiple comparisons, a *p*-value < 0.017 was considered statistically significant difference.

## 3 Results

### 3.1 Baseline characteristics of the study population

A total of 308 patients with T2DM were included in the study, of whom 125 had cognitive impairment. The incidence rate of cognitive impairment was 40.6%. The cognitive function score of T2DM patients in this study was 27.00 (25.00, 28.00). The normal cognitive function group had lower ages and female proportions compared to the cognitive impairment group. The normal cognitive function group exhibited higher education levels and a greater proportion of individuals with reading habits (*p* < 0.05). Additionally, the group with cognitive dysfunction had a longer duration of diabetes and a higher proportion of patients with peripheral neuropathy, diabetic nephropathy, hypertension, and coronary heart disease compared to the group with normal cognitive function (*p* < 0.05).In terms of biochemical indicators, the group with cognitive dysfunction had higher levels of HbA1c, FIB β2-MG, Cys-C, and GLU compared to the group with normal cognitive function. The group with cognitive dysfunction had lower levels of ALB, A/G, TBIL, IBIL, and serum sodium compared to the group with normal cognitive function (*p* < 0.05). No statistically significant differences were found in other indicators between the two groups (*p* > 0.05) (Table 1).

**Table 1:** Characteristics of patients with cognitive impairment and normal cognitive function.

Characteristics	Cognitive impairment group (*n* = 125)	Normal cognitive function group (*n* = 183)	*t/Z /χ^2^*	*p*
Age (y)	61.73 ± 9.08	58.09 ± 8.17	3.662	<0.001
Gender				
Male, *n* (%)	61 (48.8)	118 (64.5)	7.503	0.006
Female, *n* (%)	64 (51.2)	65 (35.5)		
BMI (kg/m^2^)	24.00 ± 3.63	24.61 ± 3.70	−1.431	0.153
Smoking, *n* (%)	53 (42.4)	68 (37.7)	0.684	0.408
Drinking, *n* (%)	36 (28.8)	68 (37.2)	2.320	0.128
Duration of education (y)	9 (6,12)	12 (9,15)	−4.451	<0.001
Family monthly income (yuan)			23.140	<0.001
<3,000	26 (20.8)	11 (6.0)		
3,000 ~ 5,000	73 (58.4)	97 (53.0)		
>5,000	26 (20.8)	75 (41.0)		
Exercise habits	109 (87.2)	164 (89.6)	0.431	0.512
Reading habits	7 (5.6)	23 (12.6)	4.102	0.043
SBP (mmHg)	133.09 ± 19.13	131.98 ± 17.15	0.532	0.595
DBP (mmHg)	79.02 ± 10.49	80.78 ± 12.12	−1.325	0.186
Duration of diabetes (y)	14.50 (10.00,19.00)	10.00 (5.00,15.00)	−4.712	<0.001
DPN, *n* (%)	89 (71.2)	105 (57.4)	6.087	0.014
DR, *n* (%)	16 (12.8)	19 (10.4)	0.431	0.512
DN, *n* (%)	30 (24.0)	27 (14.8)	4.210	0.040
Hypertension	61 (48.8)	68 (37.2)	4.135	0.042
CHD n (%)	30 (24.0)	24 (13.1)	6.086	0.014
Abnormal blood lipids, *n* (%)	36 (28.8)	68 (37.2)	2.320	0.128
FPG (mmol/L)	7.50 (6.73,9.43)	7.40 (6.80,8.50)	−0.902	0.367
HbA1c (%)	8.95 (7.73,10.58)	8.00 (6.90,9.20)	−4.137	<0.001
FIB (g/L)	3.00 (2.53,3.80)	2.80 (2.40,3.25)	−3.204	0.001
TG (mmol/L)	1.52 (1.14,2.26)	1.61 (1.16,2.34)	−0.678	0.498
TC (mmol/L)	4.07 (3.24,5.00)	4.08 (3.31,4.70)	−0.045	0.964
LDL-C (mmol/L)	2.21 (1.68,3.11)	2.37 (1.65,3.00)	−0.165	0.869
HDL-C (mmol/L)	0.99 (0.85,1.21)	1.01 (0.83,1.26)	−0.479	0.632
ALB (g/L)	40.98 ± 4.87	42.49 ± 4.67	−2.756	0.006
A/G	1.47 ± 0.29	1.61 ± 0.27	−4.475	<0.001
TBIL (umol/L)	10.45 (7.50,14.00)	12.60 (9.60,16.60)	−3.514	<0.001
DBIL (umol/L)	3.45 (2.10,5.00)	3.40 (2.40,4.90)	−0.431	0.666
IBIL (umol/L)	6.70 (4.95,9.28)	9.00 (6.20,12.60)	−4.309	<0.001
β2-MG (mg/L)	1.92 (1.59,2.50)	1.78 (1.45,2.12)	−2.942	0.003
Cys-C (mg/L)	0.80 (0.68,0.98)	0.71 (0.64,0.86)	−3.532	<0.001
Serum sodium (mmol/L)	141 (138,143)	142 (139,143)	−2.233	0.026
GLU (mmol/L)	8.49 (6.71,12.73)	7.62 (6.27,9.97)	−2.460	0.014

Data are presented as mean ± SDs or as number (percentage) or as median (quartile) [M (P25, P75)].

BMI, body mass index; SBP, systolic blood pressure; DBP, diastolic blood pressure; DPN, diabetic peripheral neuropathy; DR, diabetic retinopathy; DN, diabetes nephropathy; CHD, coronary heart disease; FPG, fasting plasma glucose; HbA1c, glycosylated hemoglobin; TG, triglycerides; TC, total cholesterol; LDL-C, low-density lipoprotein cholesterol; HDL-C, high-density lipoprotein cholesterol; ALB, albumin; A/G, albumin/globulin ratio; TBIL, total bilirubin; DBIL, direct bilirubin; IBIL, indirect bilirubin; Cys-C, Cystatin-C; β2-MG, β2 microglobulin; GLU, glucose; FIB, fibrinogen.

### 3.2 ROC curve analysis of continuous variables

**Figure 1:**
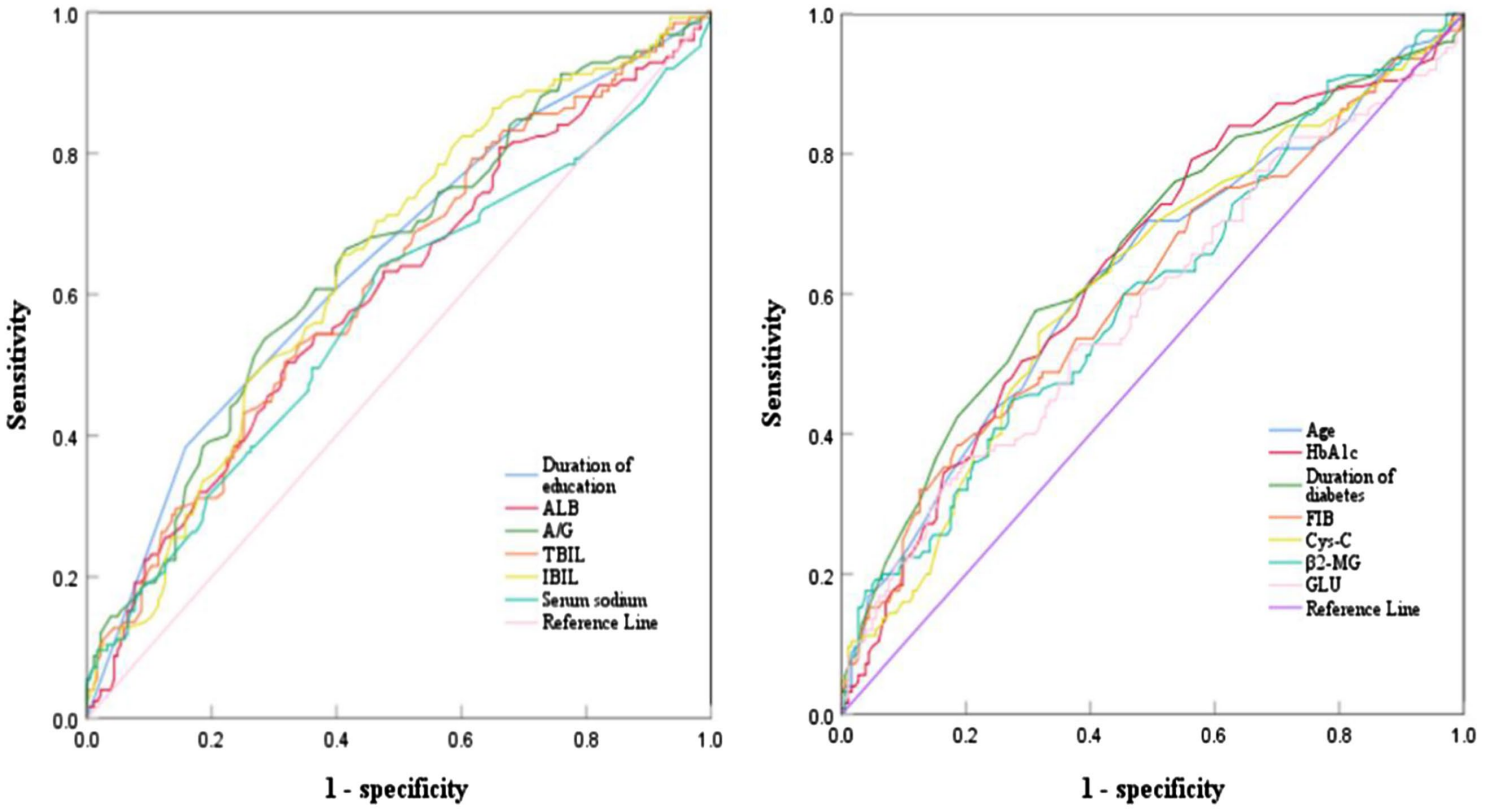
ROC curve analysis of various indicators predicting cognitive impairment in T2DM patients.

The ROC curve analysis was conducted on the continuous variables that showed statistical significance in the univariate analysis. The results indicated that age, years of education, duration of diabetes, HbA1c, FIB, ALB, A/G ratio, TBIL, IBIL, β2-MG, Cys-C, sodium, and GLU had areas under the curves of 0.626, 0.645, 0.658, 0.639, 0.607, 0.601, 0.646, 0.618, 0.645, 0.599, 0.619, 0.575, and 0583, respectively. The optimal cutoff values for these were determined as follows: 60.50 years age, 7.50 years for years of education, 13.50 years for duration of diabetes, 7.65% for HbA1c, 3.45 g/L for FIB,41.05 g/L for ALB,0.48 for A/G ratio, 10.65 μmol/L for TBIL, 8.05 μmol/L for IBIL, 2.06 mg/L for β2-MG, 0.78 mg/L for Cys-C, 141.90 mmol/L for serum sodium, and 10.64 mmol/L for GLU (Table 2, Figure 1).

**Table 2:** Results from ROC curve analysis for continuous variables.

Characteristics	AUC	Cut-point	Youden index	Sensitivity (%)	Specificity (%)
Age	0.626	60.50 years	0.220	62.40	59.60
Duration of education	0.645	7.50 years	0.226	38.40	84.20
Duration of diabetes	0.658	13.50 years	0.265	57.60	68.90
HbA1c	0.639	7.65%	0.229	79.20	43.70
FIB	0.607	3.45 g/L	0.198	38.40	81.40
ALB	0.601	41.05 g/L	0.182	50.40	67.80
A/G	0.646	1.48	0.252	53.60	71.60
TBIL	0.618	10.65 umol/L	0.189	52.80	66.10
IBIL	0.645	8.05 umol/L	0.246	65.60	59.00
β2-MG	0.599	2.06 mg/L	0.175	44.80	72.70
Cys-C	0.619	0.78 mg/L	0.227	54.40	68.30
Serum sodium	0.575	141.90 mmol/L	0.170	64.00	53.00
GLU	0.583	10.64 mmol/L	0.171	36.80	80.30

HbA1c, glycosylated hemoglobin; ALB, albumin; A/G, albumin/globulin ratio; TBIL, total bilirubin; IBIL, indirect bilirubin; Cys-C, Cystatin-C; β2-MG, β2 microglobulin; GLU, glucose; FIB, fibrinogen.

### 3.3 Analysis of factors influencing cognitive dysfunction in T2DM patients

The dependent variable was whether cognitive dysfunction occurred (0 = no, 1 = yes). Other variables such as age, family monthly income, reading habits, peripheral neuropathy, diabetes nephropathy, hypertension, coronary heart disease, HbA1c, FIB, ALB, A/G ratio, TBIL, IBIL, β2-MG, Cys-C, serum sodium, and GLU were also included in the analysis. The results indicated that female gender, duration of diabetes exceeding 13.50 years, years of education exceeding 7.50 years, and serum sodium level above 141.90 mmol/L were significant factors influencing cognitive impairment in patients with T2DM (*p* < 0.05) (Table 3).

**Table 3:** Binary logistic regression analysis of the influencing factors of cognitive dysfunction in T2DM patients.

Characteristics	β	SE	Wald	*p*	OR	95%CI
Gender						
Male	Ref					
Female	0.708	0.328	4.652	0.031	2.030	1.067 ~ 3.861
Duration of education (y)						
≤ 7.50	Ref					
> 7.50	−0.812	0.349	5.429	0.020	0.444	0.224 ~ 0.879
Duration of diabetes (y)						
≤ 13.50	Ref					
> 13.50	1.075	0.331	10.520	0.001	2.929	1.530 ~ 5.608
Serum sodium (mmol/L)						
≤ 141.90	Ref					
> 141.90	−0.741	0.337	4.822	0.028	0.477	0.246 ~ 0.923

β, regression coefficient; SE, standard error; OR, odds ratio; CI, confidence interval.

### 3.4 Medication treatment grouping situation

**Figure 2:**
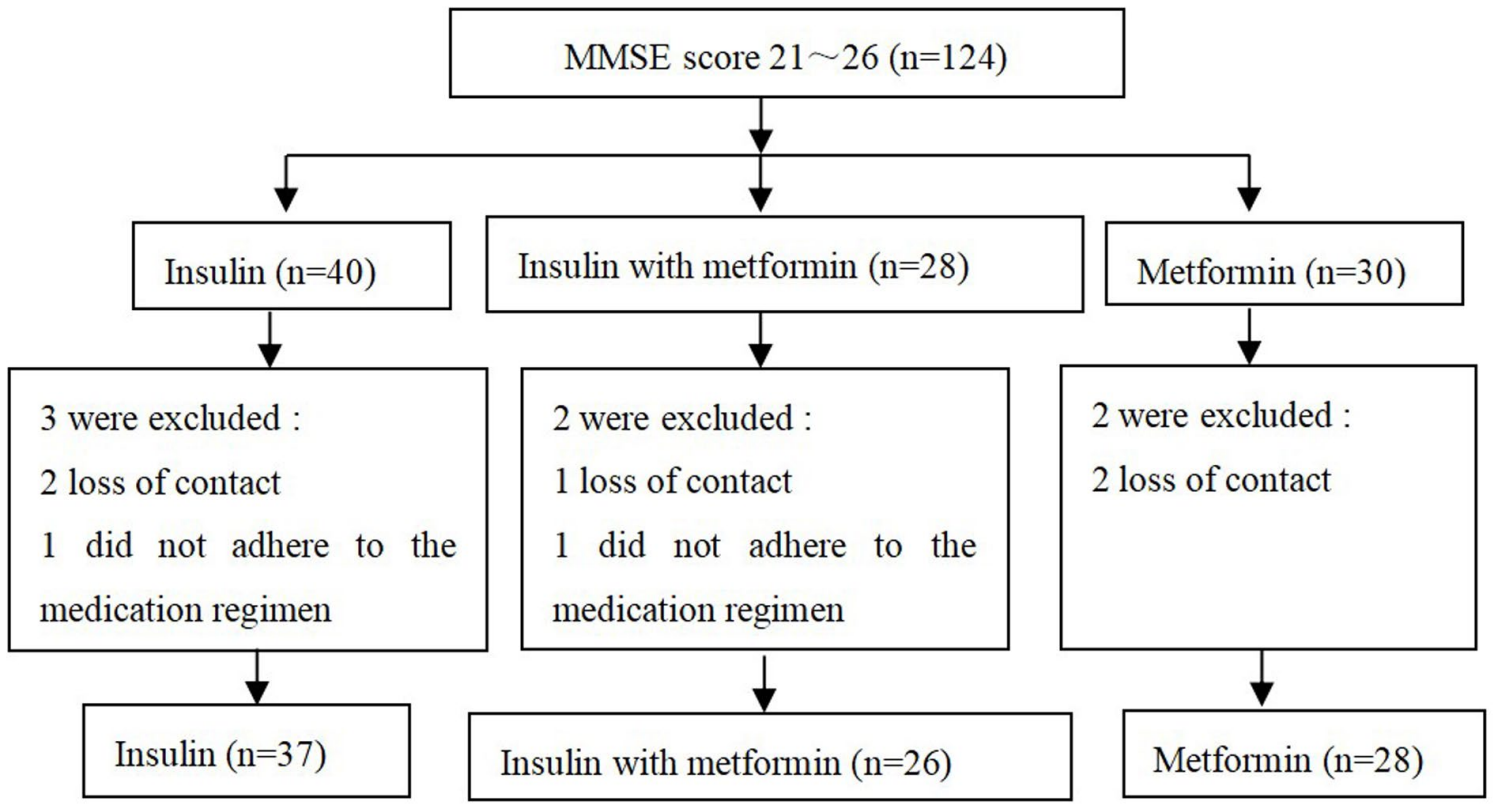
Research flowchart.

In this study, a total of 125 patients exhibited cognitive impairment, of whom 124 were diagnosed with MCI. The MCI patients were categorized into three groups based on their medication records at discharge: an insulin group consisting of 40 cases, a metformin group consisting of 30 cases, and an insulin combined with metformin group consisting of 28 cases. Two cases in the insulin group were lost to follow-up, and one case did not adhere to the medication regimen. In the metformin group, two cases were lost to follow-up. In the insulin combined with metformin group, one case was lost to follow-up, and one case did not adhere to the medication regimen. Consequently, a total of 91 patients were included in the final analysis: 37 in the insulin group, 28 in the metformin group, and 26 in the insulin combined with metformin group (Figure 2). There were no statistically significant differences in age, gender, BMI, years of education, and monthly household income among the three groups at baseline (*p* > 0.05) (Table 4).

**Table 4:** Demographic and clinical characteristics of the three patient groups.

Characteristics	Insulin group (*n* = 37)	Metformin group (*n* = 28)	Insulin with metformin group (*n* = 26)	*F/H/χ^2^*	*p*
Age (y)	60.62 ± 9.11	64.57 ± 6.86	60.50 ± 8.31	2.281	0.108
Gender				0.402	0.818
Male, *n* (%)	21 (56.8)	14 (50.0)	13 (50.0)		
Female, *n* (%)	16 (43.2)	14 (50.0)	13 (50.0)		
BMI (kg/m^2^)	22.73 ± 3.34	24.29 ± 3.50	24.16 ± 2.27	2.515	0.087
Duration of education (y)	9 (6,12)	9 (6,12)	9 (6,12)	0.177	0.915
Family monthly income (yuan)				6.889	0.142
<3,000	5 (13.5)	9 (32.1)	2 (7.7)		
3,000 ~ 5,000	22 (59.5)	14 (50.0)	15 (57.7)		
>5,000	10 (27.0)	5 (17.9)	9 (34.6)		
SBP (mmHg)	137.92 ± 17.92	134.54 ± 14.88	127.92 ± 15.83	2.840	0.064
DBP (mmHg)	78.86 ± 11.94	78.82 ± 10.00	79.85 ± 10.27	0.078	0.925
Duration of diabetes (y)	16.00 (11.50,20.00)	14.00 (10.00,18.00)	14.00 (3.50,20.25)	2.944	0.229
FPG (mmol/L)	7.80 (6.95,10.50)	7.50 (6.75,8.48)	7.35 (6.73,8.23)	2.209	0.331
HbA1c (%)	9.40 (8.35,10.45)	9.45 (8.25,10.78)	9.90 (8.15,11.95)	2.208	0.332

Data are presented as mean ± SDs or as number (percentage) or as median (quartile) [M (P25, P75)].

BMI, body mass index; SBP, systolic blood pressure; DBP, diastolic blood pressure; HbA1c, glycosylated hemoglobin; FPG, fasting plasma glucose.

### 3.5 Comparison of cognitive function before and after treatment

A comparative analysis was conducted on the cognitive function scores of three groups of patients before and after treatment. The results indicated no statistically significant difference in the total MMSE scores and scores in various dimensions among the three groups before treatment (*p* > 0.05). Following treatment, the metformin group and the insulin combined with metformin group exhibited higher MMSE total scores, attention and computational power scores, and language ability scores compared to before treatment, and these differences were statistically significant (*p* < 0.05). No such differences were observed in the scores of orientation, memory, and recall ability (*p* > 0.05). After treatment, there was no statistically significant difference (*p* > 0.05) in the total score and various dimensions of MMSE between the insulin group and before treatment. However, a statistically significant difference in MMSE total score, attention and computational power scores, and language ability scores was observed among the three groups of patients after treatment (*p* < 0.05). Both the metformin group and the insulin combined with metformin group scored higher than the insulin group, and this difference was statistically significant (*p* < 0.017). No such difference was noted between the metformin group and the insulin combined with metformin group (*p* > 0.017) (Table 5).

**Table 5:** Comparison of total MMSE scores and scores in various dimensions before and after treatment among the three patient groups.

	Group	Before treatment	After treatment	*Z*	*p*
MMSE score	Insulin	24.00 (22.00,26.00)	24.00 (22.00,26.00)	−0.296	0.831
	Metformin	24.00 (23.00,26.00)	25.00 (24.00,27.00)^*^	−3.632	<0.001
	Insulin with metformin	24.50 (24.00,26.00)	27.00 (24.00,28.00)^*^	−3.974	<0.001
	*H*	1.480	21.240		
	*P*	0.477	<0.001		
Orientation	Insulin	10.00 (9.00,10.00)	10.00 (9.00,10.00)	−0.283	0.834
	Metformin	10.00 (9.00,10.00)	10.00 (9.00,10.00)	−0.573	0.463
	Insulin with metformin	10.00 (9.00,10.00)	10.00 (10.00,10.00)	−1.356	0.176
	*H*	1.053	4.565		
	*P*	0.591	0.107		
Memory	Insulin	3.00 (3.00,3.00)	3.00 (3.00,3.00)	−0.162	0.987
	Metformin	3.00 (3.00,3.00)	3.00 (3.00,3.00)	−1.342	0.180
	Insulin with metformin	3.00 (3.00,3.00)	3.00 (3.00,3.00)	−0.287	0.845
	*H*	4.550	4.675		
	*P*	0.103	0.092		
Attention, calculation	Insulin	3.00 (2.00,3.00)	3.00 (2.00,3.00)	−0.192	0.932
	Metformin	3.00 (2.00,3.75)	3.50 (3.00,4.00)^*^	−2.919	0.004
	Insulin with metformin	3.00 (2.00,3.00)	4.00 (3.00,4.00)^*^	−3.581	<0.001
	*H*	0.576	16.382		
	*P*	0.750	<0.001		
Recall	Insulin	2.00 (2.00,2.00)	2.00 (2.00,2.00)	−0.256	0.876
	Metformin	2.00 (2.00,2.00)	2.00 (2.00,2.00)	−0.302	0.763
	Insulin with metformin	2.00 (2.00,2.00)	2.00 (2.00,3.00)	−0.333	0.739
	*H*	1.888	4.035		
	*P*	0.389	0.133		
Language	Insulin	7.00 (6.00,7.00)	7.00 (6.00,7.00)	−0.267	0.856
	Metformin	7.00 (6.00,7.75)	7.50 (7.00,8.00)^*^	−2.364	0.018
	Insulin with metformin	7.00 (6.00,7.00)	8.00 (7.00,8.00)^*^	−3.124	0.002
	*H*	0.701	15.705		
	*P*	0.704	<0.001		

Compared with the insulin group, *p < 0.017.

## 4 Discussion

In this study, the cognitive impairment rate of T2DM patients was 40.6%. Female gender, duration of diabetes exceeding 13.50 years, years of education exceeding 7.50 years, and serum sodium level above 141.90 mmol/L were significant factors influencing cognitive impairment in patients with T2DM. Metformin and insulin combined with metformin might improve cognitive function in T2DM patients with MCI, and the effect is superior to insulin alone. No differences in the therapeutic effects of metformin and insulin combined with metformin were observed. The findings of this research have significant implications for screening cognitive impairment in T2DM patients, as well as early detection and treatment. Clarifying the therapeutic effects of hypoglycemic drugs on MCI patients is beneficial for targeted medication and improving treatment effectiveness, which is of great significance in preventing senile dementia and delaying the progression of dementia.

### 4.1 Current status of cognitive dysfunction in T2DM patients

The results of this study indicate that the cognitive dysfunction rate among T2DM patients is 40.6%, which is lower than the prevalence rate (48%) calculated by Chen et al. (6). Chen’s research concentrated on elderly diabetic patients, and age is considered a risk factor for cognitive impairment in T2DM patients. In this study, the participants were T2DM patients of various ages, resulting in a lower cognitive dysfunction rate than in Chen’s study. Furthermore, the cognitive function score of T2DM patients in this study was 27.00 (25.00, 28.00), suggesting a good overall cognitive function. It is important to note that the MMSE scale, used to assess cognitive function, may not be sensitive enough to detect MCI, leading to higher specificity but lower sensitivity compared to the Montreal Cognitive Assessment. Therefore, future research is advised to utilize a combination of both scales to enhance the accuracy of diagnosing cognitive impairment.

### 4.2 Factors influencing cognitive dysfunction in T2DM patients

#### 4.2.1 Gender

The results of this study suggest that female have a significantly higher risk of developing cognitive impairment than men with T2DM (OR = 2.030, 95% CI: 1.067–3.861), which is consistent with the findings of Verhagen et al. (19). Giacomucci et al. (20) propose that estrogens have neuroprotective effects; however, a decline in estrogen levels during menopause may contribute to cognitive decline. The majority of women in this study are middle-aged and elderly, within the premenopausal and menopausal periods, which may contribute to cognitive impairments due to reduced hormone levels. Research has shown that women carrying the APOE4 variant gene have a fourfold higher risk of developing AD than men carrying the same gene, while the APOE4 gene has a minimal impact on men (21). The higher risk of cognitive impairment in women may be attributed to the presence of the APOE4 gene. Additionally, the education level of women in this study was lower than that of men. This shorter duration of education may also contribute to the higher risk of cognitive impairment in women. Therefore, it is essential to pay more attention to the cognitive function of women in clinical practice, conduct regular cognitive screening, and provide early detection and intervention for cognitive dysfunction.

#### 4.2.2 Duration of education

The study indicates that individuals with T2DM and an educational duration of over 7.50 years have a significantly lower risk of cognitive impairment than those with an educational duration of less than or equal to 7.50 years (OR = 0.444, 95% CI: 0.224–0.879). One possible explanation for this phenomenon is that individuals with higher levels of education, such as knowledge workers, tend to have a higher synaptic density in the cerebral cortex. As a result, their brains have increased storage capacity and are able to delay the onset of dementia symptoms by approximately 4–5 years (8). A cross-sectional study involving 1,023 participants found that individuals with formal education exhibited better cognitive function and a lower risk of dementia than those without education (22). Some researchers believe that education has a positive impact on cognitive function in individuals aged 50 and above and may even counteract the negative effects of low-income living on cognitive health (23). This finding aligns with Sun et al.’s research (8), which suggests that a higher level of education acts as a protective factor against cognitive dysfunction in T2DM patients. Furthermore, this study further reveals that an educational duration exceeding 7.50 years significantly reduces the risk of cognitive impairment in patients. Healthcare professionals should pay attention to T2DM patients with an educational duration of less than or equal to 7.50 years, conduct regular cognitive function assessments, and gain a comprehensive understanding of their cognitive health.

#### 4.2.3 Duration of diabetes

The study results also indicated that among T2DM patients, those with a diabetes duration exceeding 13.50 years exhibited a significantly elevated risk of cognitive dysfunction compared to patients with a diabetes duration of ≤13.50 years (OR = 2.929, 95% CI: 1.530–5.608). This may be attributed to impaired peripheral insulin action leading to hyperglycemia, which in turn causes vascular damage, glucose neurotoxicity, and an increased risk of dementia due to the accumulation of advanced glycation end products (24). Moreover, T2DM patients with a disease duration of over 20 years have a significantly higher risk of cognitive impairment, particularly in processing speed and attention (25). The study found that the cognitive decline of T2DM patients is positively correlated with the duration of diabetes. Patients diagnosed for more than 15 years have a significantly increased risk of cognitive dysfunction (8), which aligns with the findings of this study. Therefore, in clinical practice, medical professionals should be vigilant when the duration of diabetes exceeds 13.50 years and proactively identify and intervene in cases of cognitive impairment.

#### 4.2.4 Serum sodium

Furthermore, the study results revealed that among T2DM patients, those with a serum sodium concentration greater than 141.90 mmol/L had a significantly reduced risk of cognitive impairment compared to patients with a serum sodium concentration of ≤141.90 mmol/L (OR = 0.477, 95% CI: 0.246–0.923). Hyponatremia refers to a pathological condition where the serum sodium concentration is less than 135 mmol/L. The cognitive impairment observed in these cases may be attributed to the activation of the renin-angiotensin system, induction of mitochondrial dysfunction and oxidative stress, and decreased ATP production in hippocampal cells. Studies have demonstrated that compared to the control group, individuals with hyponatremia exhibit significantly poorer scores across various domains of cognitive function (26). Researchers have also noted that elevated sodium levels independently predict improvements in MMSE cognitive function scores, with the resolution of hyponatremia positively impacting the overall cognitive function of elderly patients (27). Van der Burgh et al. (28) pointed out that low serum sodium levels, even within the range above the clinical threshold of hyponatremia, are associated with cognitive impairments in attention and psychomotor function, confirming the results of this study. Building on these findings, the study further revealed that a serum sodium concentration greater than 141.90 mmol/L significantly reduces the risk of cognitive impairment in patients. However, it is important to note that excessive serum sodium concentration can also negatively impact cognitive abilities, suggesting the presence of an optimal serum sodium concentration range. Consequently, further research is necessary to pinpoint this threshold.

### 4.3 Comparison of treatment effects in patients with T2DM accompanied by MCI

The study findings suggest that following a 6-month medication regimen, both the metformin group and the insulin-combined metformin group experienced enhancements in total MMSE scores, as well as attention and computational abilities, and language abilities relative to their baseline levels. Cardoso and Moreira (29) has posited that metformin might bolster executive function, learning and memory, and cognitive attention in AD patients, in line with the present study’s observations. This improvement is possibly attributed to metformin’s capacity to stimulate adult hippocampal neurogenesis, deter amyloid plaque formation, restore normal insulin signaling within neural cells, and ameliorate pathological alterations in neural lines subjected to chronic high-insulin conditions (29). The central nervous system’s insulin can decrease tau protein phosphorylation rates, suppress microglial activation, and regulate cognitive and memory functions by tweaking the synthesis of anti-inflammatory mediators and pro-inflammatory factors (29). The combined therapy may elicit a synergistic impact, resulting in higher scores across various dimensions, including MMSE, attention, computational power, and language abilities, post-treatment compared to pre-treatment.

The study found no significant differences between the metformin group and the insulin combined with metformin group in terms of orientation, memory, and recall abilities before and after treatment. This may be due to the relatively high baseline scores for these dimensions in patients, indicating that these functions were not severely impaired. Additionally, the insulin group did not show any changes in total MMSE score or other dimensional scores before and after treatment, suggesting that insulin alone may not have a significant impact on cognitive function in MCI patients. In contrast, other studies have reported improvements in cognitive function and glucose metabolism in patients with MCI or AD when intranasal insulin therapy was used (30). Hallschmid’s study (11) also showed improved story recall ability after 4 months of treatment with regular insulin. The discrepancies between these studies and the current one may be attributed to two reasons: (1) The subcutaneous injection of insulin in this study has limitations, as it can easily lead to hypoglycemia and increase the risk of cognitive impairment in patients. Moreover, the blood–brain barrier may impede insulin transport, rendering it ineffective. (2) Considering the sample size, this study included two types of insulin, Mendong and Degu, in the insulin group. Each patient had different medication doses, which could contribute to the variations observed between this study and others (31).

Previous research has also shown that combining metformin with donepezil can improve cognitive function and glucose metabolism abnormalities in patients (32). Dahl et al.’s study (33) revealed that subcutaneous injection of tigapamide in addition to adjusting insulin glargine can significantly reduce blood sugar levels. These studies suggest that combination therapy may yield better outcomes. However, our study did not find significant differences between the metformin group and the insulin combined with metformin group in terms of the total MMSE score and other dimensional scores after treatment. This finding might be associated with the negative impact of hypoglycemia caused by subcutaneous insulin injection on cognitive function and the blood–brain barrier. Such effects can lead to reduced insulin absorption and utilization, thereby compromising its effectiveness.

### 4.4 Advantages and limitations

This study offers several strengths. First, it comprehensively assesses various relevant factors influencing cognitive dysfunction in T2DM patients. By calculating the optimal clinical cutoff points for each risk factor through ROC curve analysis based on previous studies, a multifactor analysis was conducted. This method ensures that the findings are more targeted and beneficial for screening T2DM patients with cognitive impairment. Second, while insulin and metformin have been shown to improve cognitive function in T2DM patients, few studies compare their therapeutic effects. This study is among the few to make such a comparison. However, our study also has limitations that should be acknowledged. First, monitoring and controlling medication adherence post-discharge posed a challenge. Adherence was assessed via self-report, potentially introducing bias. Second, there was a lack of long-term evaluation of hypoglycemic drugs’ effects on cognitive impairment. Initially, short-term and long-term evaluations at 6 and 12 months post-discharge were planned, but due to time constraints and some patients being readmitted before the second evaluation, discharge medication records were altered. Consequently, the data for 12 months after discharge was not included. In future research, a comprehensive consideration of objective conditions should be made to develop a feasible research plan and examine both short-term and long-term effects.

Future studies could conduct large-sample, multicenter randomized controlled trials to explore the impact of different dosages and administration methods on clinical efficacy, identifying the optimal dosage and administration method for treating T2DM patients with cognitive impairment.

## 5 Conclusion

In summary, this study found a 40.6% prevalence of cognitive impairment in T2DM patients. Factors associated with cognitive dysfunction included female gender, diabetes duration exceeding 13.50 years, education duration over 7.50 years, and serum sodium levels above 141.90 mmol/L. Metformin and insulin combined with metformin were effective in improving cognitive function in T2DM patients with MCI, with a superior effect compared to insulin alone. No significant differences in therapeutic effects were observed between the metformin group and the insulin combined with metformin group.

## Data availability statement

The raw data supporting the conclusions of this article will be made available by the authors, without undue reservation.

## Ethics statement

The studies involving humans were approved by Ethics Committee of Yan’an University Affiliated Hospital. The studies were conducted in accordance with the local legislation and institutional requirements. The participants provided their written informed consent to participate in this study. Written informed consent was obtained from the individual(s) for the publication of any potentially identifiable images or data included in this article.

## Author contributions

ML: Data curation, Investigation, Writing – original draft. ZW: Funding acquisition, Resources, Writing – review & editing. JH: Conceptualization, Writing – review & editing. ZM: Investigation, Writing – original draft. HB: Conceptualization, Writing – review & editing.
